# Urinary MicroRNA-21-5p as Potential Biomarker of Interstitial Fibrosis and Tubular Atrophy (IFTA) in Kidney Transplant Recipients

**DOI:** 10.3390/diagnostics10020113

**Published:** 2020-02-19

**Authors:** Michal S. Gniewkiewicz, Izabela Paszkowska, Jolanta Gozdowska, Katarzyna Czerwinska, Anna Sadowska-Jakubowicz, Dominika Deborska-Materkowska, Agnieszka Perkowska-Ptasinska, Maciej Kosieradzki, Magdalena Durlik

**Affiliations:** 1 Department of Transplantation Medicine, Nephrology and Internal Medicine, Medical University of Warsaw, Nowogrodzka 59, 02-006 Warsaw, Poland; michal.gniewkiewicz@gmail.com (M.S.G.); pas.izaaa@gmail.com (I.P.); k.przychodzka@onet.eu (K.C.); anna.sadowska-jakubowicz@wum.edu.pl (A.S.-J.); dominika.deborska-materkowska@wum.edu.pl (D.D.-M.); agnieszka.perkowska-ptasinska@wum.edu.pl (A.P.-P.); magdalena.durlik@wum.edu.pl (M.D.); 2 Department of General and Transplantation Surgery, Medical University of Warsaw, Nowogrodzka 59, 02-006 Warsaw, Poland; maciej.kosieradzki@wum.edu.pl

**Keywords:** IFTA, kidney transplantation, microRNA, miR-21

## Abstract

Chronic renal allograft dysfunction (CAD) is a major limiting factor of long-term graft survival. The hallmarks of progressive CAD are interstitial fibrosis and tubular atrophy (IFTA). MicroRNAs are small, regulatory RNAs involved in many immunological processes. In particular, microRNA-21-5p (miR-21) is considered to be strongly associated with pathogenesis regarding tubulointerstitium. The aim of this study was to assess urinary miR-21 expression levels in the kidney transplant recipients and determine their application in the evaluation of IFTA and kidney allograft function. The expression levels of miR-21 were quantified in the urine of 31 kidney transplant recipients with biopsy-assessed IFTA (IFTA 0 + I: *n* = 17; IFTA II + III: *n* = 14) by real-time quantitative PCR. Urine samples were collected at the time of protocolar biopsies performed 1 or 2 years after kidney transplantation. MicroRNA-191-5p was used as reference gene. MiR-21 was significantly up-regulated in IFTA II + III group compared to IFTA 0 + I group (*p* = 0.003). MiR-21 correlated significantly with serum concentration of creatinine (r = 0.52, *p* = 0.003) and eGFR (r = −0.45; *p* = 0.01). ROC analysis determined the diagnostic value of miR-21 with an area under curve (AUC) of 0.80 (*p* = 0.0002), sensitivity of 0.86 and specificity of 0.71. miR-21 is associated with renal allograft dysfunction and IFTA. Therefore, it could be considered as a potential diagnostic, non-invasive biomarker for monitoring renal graft function.

## 1. Introduction

Kidney transplantation (KTx) is currently the treatment of choice for patients with end-stage renal disease (ESRD). In comparison to dialysis, KTx prolongs the survival of patients, increases their quality of life and is more cost-effective. Within the last few decades, short graft survival has significantly improved due to modern immunosuppressive agents, advances in surgical techniques, organ preservation and postoperative care. Despite these contributions, similar improvement in long-term outcomes was not observed [1].

Chronic renal allograft dysfunction (CAD) is one of the leading causes of long-term graft survival [2]. Usually it is clinically asymptomatic and begins after the first transplantation year, resulting in continuous decline in allograft function [3]. Early detection of this process is crucial as persistent dysfunction without appropriate intervention may lead to irreversible damage to the allograft and, eventually, allograft loss [4]. The greatest hallmarks of progressive CAD are interstitial fibrosis and tubular atrophy (IFTA) [5]. IFTA could be classified as mild (grade I, <25% of cortical area), moderate (grade II, 25–50%) and severe (grade III, >50%) according to the Banff grading system [6].

Processes at the molecular level involved in chronic allograft injury occur very early, but they do not manifest clinically and cannot be detected by standard methods [7]. Currently, assessment of IFTA is only possible with renal biopsy, which is an invasive procedure [8]. Postoperative management of kidney transplant recipients includes protocolar biopsies which are performed without specific clinical indications to detect subclinical changes [9]. They are considered to be safe with a major complications rate of 0–4% (demanding surgical intervention, blood transfusion or prolonged hospitalization) in various cohorts [10]. However, renal biopsy can be contraindicated in some patients and cannot be performed repetitively [11].

Early molecular diagnosis of IFTA would allow one to initiate appropriate treatment options well in advance to prevent further allograft injury [12]. A non-invasive, easily accessible biomarker that could assess the status of IFTA and predict the progression of dysfunction is needed [13].

MicroRNAs (miRNAs) represent a class of small, endogenous, noncoding RNAs which target complementary sites of messenger RNA (mRNA) and regulate their expression [14]. They are involved in various physiological and pathological processes including cell proliferation, differentiation, apoptosis, fibrosis and inflammation, as well as angiogenesis and oncogenesis [15,16].

Due to small size, stability and presence in body fluids miRNAs have the potential to act as biomarkers and therapeutic targets. They could be inactivated by antagomirs or serve as therapeutic agents as locked nucleic acids. The research field is constantly expanding and the clinical application of miRNAs in KTx is widely evaluated [17].

Particularly, miR-21 seems to contribute strongly to the pathogenesis of tubulointersitital fibrosis [18]. MiR-21 was widely analyzed in many pathological conditions of native kidneys, such as diabetic nephropathy, IgA nephropathy, autosomal dominant polycystic kidney disease (ADPKD) or renal cell carcinoma [19,20,21,22,23]. There are few studies related to renal transplantation outcomes describing the role of miR-21. Available research suggests that higher expression of miR-21 is associated with fibrosis and kidney allograft injury [24,25].

The aim of this study was to assess urinary miR-21 expression levels in the kidney transplant recipients and to determine their application in the evaluation of IFTA and renal allograft function.

## 2. Materials and Methods

### 2.1. Study Population

Thirty-one patients who underwent kidney transplantation from brain-dead deceased donors between 2016 and 2018 were included in the study. A graft protocolar biopsy procedure was performed one or two years after KTx in all patients. During biopsy, patients were in good health, without any symptoms of acute infection, the C-reactive protein was within the normal range and the kidney allograft function was stable. The biopsy was done using an 18-gauge needle with ultrasound guidance. The biopsy specimens were evaluated based on Banff criteria by a single nephropathologist [6]. According to the biopsy results, patients were categorized into two groups: IFTA 0 + I (*n* = 17) and IFTA II + III (*n* = 14). IFTA 0 means no IFTA was present. IFTA I was combined with IFTA 0 as it represents the natural course of transplant lesions in renal allograft [26]. Histopathological examination does not reveal any other significant findings as rejection, recurrence of native kidney disease or BK virus nephropathy. All participants were Caucasians and were on triple immunosuppressive therapy (tacrolimus, mycophenolate mofetil and corticosteroids).

The study was conducted in accordance with the Declaration of Helsinki and was approved by the medical ethics committee of the Medical University of Warsaw. Informed written consent was obtained from all patients.

### 2.2. MicroRNA Analysis

Urine single spot samples were collected at the day of biopsy and stored at −80 °C until miRNA extraction. MagMAX mirVana Total RNA Isolation Kit (Applied Biosystems, Thermo Scientific, Vilnius, Lithuania) with MagMAX magnetic bead technology was used to extract miRNA from urine samples according to the manufacturer’s protocol. The extracted miRNA was reverse transcribed into complementary DNA (cDNA) using the TaqMan Advanced miRNA cDNA Synthesis Kit (Applied Biosystems, Thermo Scientific, Vilnius, Lithuania).

Real-time quantitative polymerase chain reaction (qPCR) was performed to quantify miR-21 and microRNA-191-5p (miR-191) using TaqMan Advanced miRNA Assay and TaqMan Fast Advanced Master Mix (Applied Biosystems, Thermo Scientific, Vilnius, Lithuania). TaqMan microRNA assays IDs were 477975_mir (hsa-miR-21-5p) and 477952_mir (hsa-miR-191-5p).

MiR-191 was used for normalization as it is described as a stable gene showing a low degree of variation and high stability value [27,28]. The relative gene expression levels were expressed by the ΔCt values (ΔCt = Ct_miR-21_ − Ct_miR191_, where Ct is the cycle threshold value) [29]. Lower ΔCt values indicate a higher relative expression of the miR-21. All experiments were carried out in duplicate and the arithmetic mean value was used for analysis.

### 2.3. Statistical Analysis

Normality of distribution was assessed by the Shapiro Wilk-test. Categorical data were described as a number (percentage) and continuous data were expressed as mean values with standard deviations (SD) or medians with quartiles 1 and 3 (Q1, Q3). Chi square or the Fisher Exact test were used to compare the categorical variables. Continuous data were compared by 2-sample t tests (parametric) or the Mann–Whitney test (non-parametric). Correlations were evaluated using the Pearson rank correlation coefficient. In order to determine the possibility of the application of miR-21 as a marker of IFTA II-III, a Receiver Operating Characteristic (ROC) curve was performed. Area under curve (AUC), sensitivity and specificity with 95% confidence intervals (CI) were determined. A *p* value < 0.05 was considered significant.

## 3. Results

Thirty-one kidney transplant recipients from our transplant center were included in the study. Based on the biopsy results, the patients were divided into groups: IFTA 0 + I (*n* = 17) and IFTA II + III (*n* = 14).

Table 1 depicts the characteristics of the groups. There was no statistical difference between the groups regarding age at biopsy, sex, body mass index, whether it was their first transplantation, cold and warm ischemia times, incidence of delayed graft function, time of biopsy after KTx, human leukocyte antigen (HLA) mismatches, immunosuppressive therapy and blood concentration of tacrolimus at the time of biopsy. Patients in the IFTA II + III group had significantly higher serum concentrations of creatinine and a lower estimated glomerular filtration rate (eGFR) (calculated using the CKD-EPI creatinine equation).

**Figure 1:**
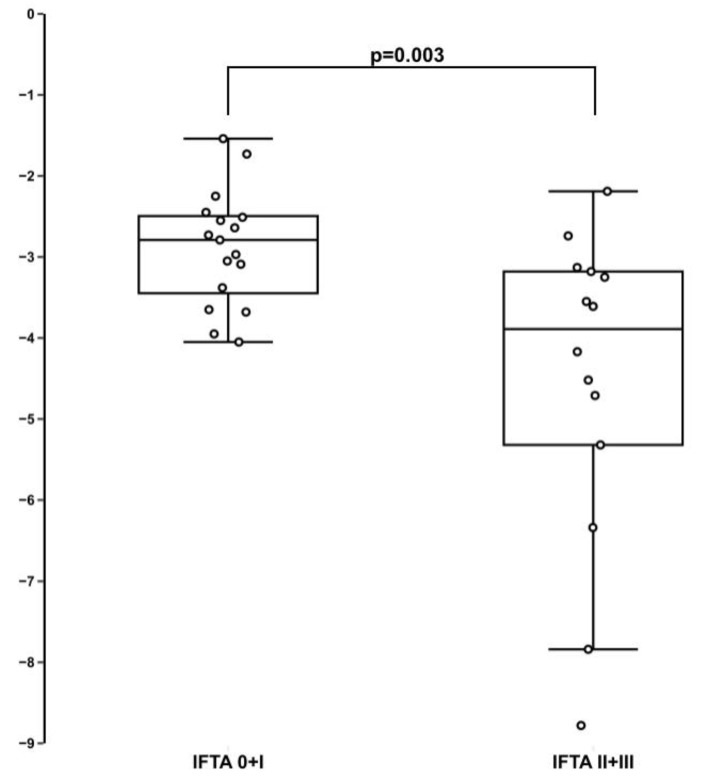
The relative expression levels of miR-21 in the IFTA 0 + I group compared to the IFTA II + III group. Small circle represents a single data point.

MiR-21 was quantified in the patients’ urine using qPCR. The relative expression of miR-21 normalized to miR-191 (ΔCt = Ct_miR-21_ − Ct_miR-191)_ is shown in Figure 1. Significantly increased levels of miR-21 was observed in the IFTA III + II group compared to the IFTA 0 + I group (−4.52 versus −2.88, *p* = 0.003).

**Figure 2:**
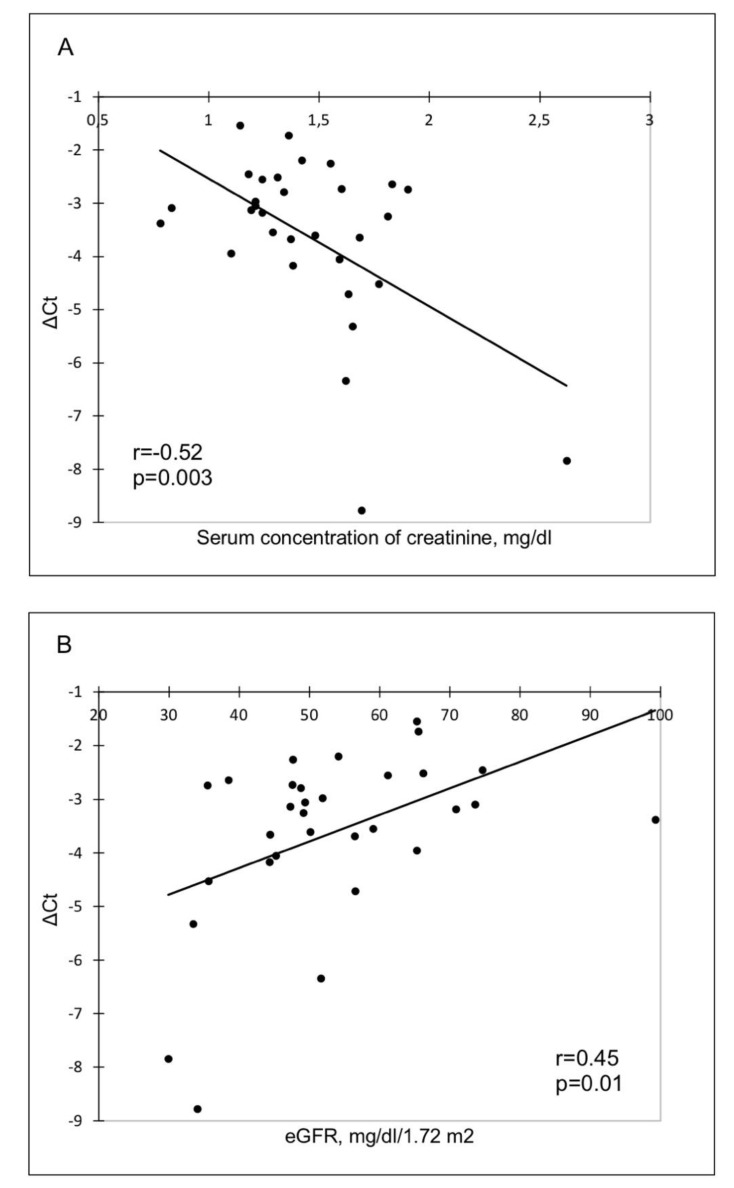
Correlation of miR-21 with (**A**) serum concentration of creatinine and (**B**) eGFR.

The relationship between the ΔCt value and clinical parameters in the study population was evaluated. A statistically significant negative correlation of the ΔCt value with serum concentration of creatinine (*r* = −0.52, *p* = 0.003) and a positive correlation with eGFR (*r* = 0.45; *p* = 0.01) were found (Figure 2).

**Figure 3:**
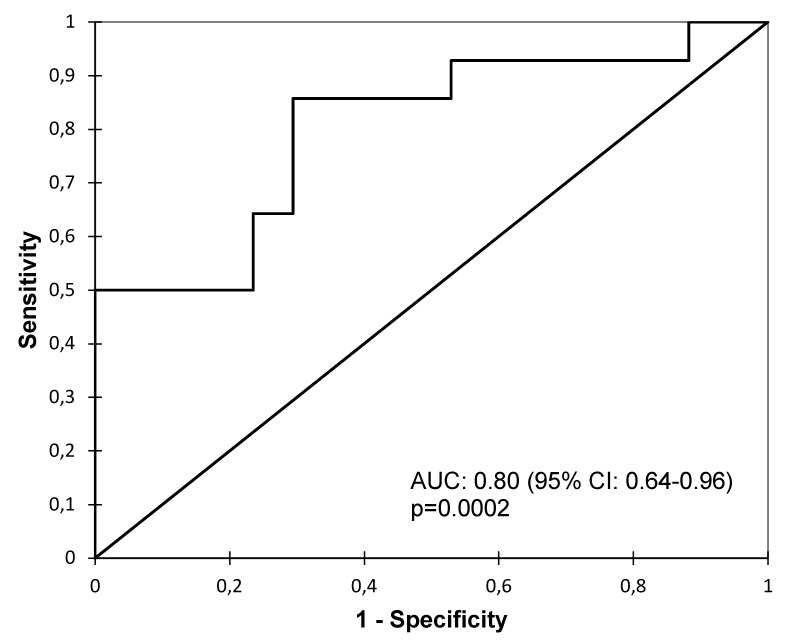
The ROC curve analysis of miR-21to discriminate between the IFTA 0 + I and IFTA II + III groups.

The ROC analysis was performed to assess whether the urine level of miR-21 could distinguish the IFTA II + III group from the IFTA 0 + I group (Figure 3). The analysis revealed a good diagnostic value of miR-21 with an AUC of 0.80 (95% CI: 0.64–0.96, *p* = 0.0002), sensitivity of 0.86 (95% CI: 0.59–0.97) and specificity of 0.71 (95% CI: 0.47–0.87).

## 4. Discussion

There is a great need in transplantation medicine for novel, non-invasive biomarkers that allow one to identify allograft dysfunction at an early stage, of which may eventually replace transplant biopsy as a gold standard. Urine is a type of preferred specimen regarding kidney diseases as it contains biochemical and cellular elements derived directly from glomerular filtration of plasma, excretion of renal tubules and secretion of the urinary tract, which indicate pathophysiology and metabolism of an individual at a certain time. Urine can be collected repeatedly in a completely non-invasive manner and in a relatively large volume. Possible clinical applications of different urinary biomarkers including miR-21 are widely tested [30,31].

In the present study, we analyzed urinary miR-21 as a biomarker reflecting renal allograft function and histopathology (IFTA). Significantly higher expression of miR-21 was observed in patients with moderate and severe IFTA (≥25% of cortical area of kidney allograft) and worse allograft function. MiR-21 was moderately positively correlated with the serum concentration of creatinine and negatively with eGFR. Previous studies reported similar outcomes. Khalid et al. showed that urinary miR-21 was significantly upregulated in the patients with delayed graft function in the days following renal transplantation [24]. Ben-Dov et al. revealed that miR-21 expression is increased in the tissue samples of human kidney allografts diagnosed with IFTA [32].

Pro-fibrotic potential of miR-21 was studied in animal models. It was shown that miR-21 in the murine kidney is a post-transcriptional regulator that enhances kidney injury and as a result, increases renal fibrosis. Notably, the PPARα-regulated metabolic signaling pathway (TGF-β1/Smad3 pathway), which is reported to be highly protective against the development of fibrosis, is silenced by miR-21 [33,34]. In a recent study, Sun et al. suggested that the antifibrotic effect of pioglitazone, which is a type of PPARγ agonist, is connected with the modulation of the miR-21-5p/Smad-7 pathway [35]. Tacrolimus-induced nephropathy (which especially manifests as IFTA or glomerular sclerosis) was associated with upregulation of miR-21 in mice renal proximal tubular epithelial cells [36]. Other mechanisms of promoting kidney fibrosis involving miR-21 are also described in the literature [37,38,39].

To determine the diagnostic value of urinary miR-21 as a marker of moderate and severe IFTA, ROC curve analysis was performed. MiR-21 exhibits good diagnostic accuracy with AUC of 0.8, 86% sensitivity and 71% specificity. Zununi Vahed et al. presented comparable results indicating not only strong potential of miR-21, but also of miR-200b in the diagnosis of IFTA [40]. Analogous studies were conducted regarding the expression of circulating miR-21, confirming the pathogenic character of miR-21 and its association with allograft fibrosis [18,25].

Our results are consistent with the available literature. However, study groups differ significantly in their relative expression of miR-21 and eGFR, so it cannot be clearly concluded that miR-21 better reflects the IFTA status of the patient than eGFR. Large randomized trials should be planned to assess the utility of miR-21 in routine clinical practice. The study is single centre with a limited number of participants, which is mainly due to rigorous inclusion criteria. Furthermore, it is important to evaluate the role of anti-miR-21 agents in preventing kidney fibrosis. This may provide an additional way of managing CAD in kidney transplant recipients.

The study has confirmed that urinary miR-21 is associated with renal allograft dysfunction and IFTA. Therefore, it could be considered as a potential diagnostic, non-invasive biomarker for monitoring renal allograft function and the development of IFTA.

## Figures and Tables

**Table 1:** Characteristics of the study groups.

	IFTA 0 + I (*n* = 17)	IFTA II + III (*n* = 14)	*p* Value
Age at biopsy, years, mean (SD)	54.76 (10.74)	48.38 (14.95)	0.18
Male sex, *n* (%)	12 (70.59)	8 (57.14)	0.31
Body mass index, kg/m^2^, mean (SD)	25.48 (3.48)	23.58 (4.06)	0.18
First transplantation, *n* (%)	15 (88.24)	14 (100.00)	0.49
Cold ischemia time, min, mean (SD)	1249.33 (420.32)	1262.14 (530.34)	0.95
Warm ischemia time, min, mean (SD)	35.08 (7.47)	32.08 (5.33)	0.25
Delayed graft function, *n* (%)	5 (29.41)	5 (35.71)	0.71
Time of biopsy after transplantation			0.82
1 year, *n* (%)	9 (52.94)	8 (57.14)	
2 years, *n* (%)	8 (47.06)	6 (42.86)	
Serum creatinine, mg/dl, mean (SD)	1.31 (0.28)	1.62 (0.35)	0.01
eGFR (CKD-EPI), mg/dl/1.72 m^2^, mean (SD)	58.82 (14.99)	46.50 (11.77)	0.02
Human leukocyte antigen (HLA) mismatches, mean (Q1–Q3)	3.59 (2.50–5.00)	3.29 (3.00–4.00)	0.52
Immunosuppressive therapy (tacrolimus, mycophenolate mofetil and corticosteroids), *n* (%)	17 (100)	14 (100)	1.00
Blood tacrolimus level, ng/mL, mean (SD)	6.02 (1.79)	5.72 (1.43)	0.48
